# Recent global decline in rainfall interception loss due to altered rainfall regimes

**DOI:** 10.1038/s41467-022-35414-y

**Published:** 2022-12-10

**Authors:** Xu Lian, Wenli Zhao, Pierre Gentine

**Affiliations:** 1 Department of Earth and Environmental Engineering, Columbia University, New York, NY USA; 2 Center for Learning the Earth with Artificial intelligence and Physics (LEAP), Columbia University, New York, NY USA

**Keywords:** Hydrology, Ecology

## Abstract

Evaporative loss of interception (*E*_*i*_) is the first process occurring during rainfall, yet its role in large-scale surface water balance has been largely underexplored. Here we show that *E*_*i*_ can be inferred from flux tower evapotranspiration measurements using physics-informed hybrid machine learning models built under wet versus dry conditions. Forced by satellite and reanalysis data, this framework provides an observationally constrained estimate of *E*_*i*_, which is on average 84.1 ± 1.8 mm per year and accounts for 8.6 ± 0.2% of total rainfall globally during 2000–2020. Rainfall frequency regulates long-term average *E*_*i*_ changes, and rainfall intensity, rather than vegetation attributes, determines the fraction of *E*_*i*_ in gross precipitation (*E*_*i*_/*P*). Rain events have become less frequent and more intense since 2000, driving a global decline in *E*_*i*_ (and *E*_*i*_/*P*) by 4.9% (6.7%). This suggests that ongoing rainfall changes favor a partitioning towards more soil moisture and runoff, benefiting ecosystem functions but simultaneously increasing flood risks.

## Abstract (web summary)

Canopy rainfall interception (*E*_*i*_) is a key component of global water cycle. Here, the authors quantify *E*_*i*_ using flux tower data and machine learning, and find that rainfall gets less partitioned into *E*_*i*_ as it gets more intense and less frequent.

## Introduction

Over vegetated landscapes, rainfall is first intercepted and temporally stored on leaves, branches, stems, lichens, or litter on the forest floor, much of which is subsequently recycled to the atmosphere^1–3^. This evaporative loss of intercepted rainfall (*E*_*i*_), buffers rainfall intensity, redistributes surface available water, and provides rapid moisture feedbacks to the atmospheric water cycle^4^. Leaving this part of rainfall aside, the remaining water reaches the soil surface through throughfall and stemflow, recharging the soil reservoir or running off into streams/rivers. In this regard, the amount of *E*_*i*_ directly influences how much water will be available in the soil for sustaining vegetation growth and functioning, which is important especially in water-stressed regions/seasons. Observational evidence also shows that any bias in this *E*_*i*_ flux would be directly propagated to estimates of key eco-hydrological parameters such as the ratio of plant transpiration to total evapotranspiration (ET)^5–7^. Therefore, a better understanding of canopy rainfall interception will shed light into other hydrological fluxes involved in the precipitation-to-runoff processes, and help better constrain the ecosystem water availability and actual ecosystem water use.

*E*_*i*_ is usually measured at the site level as the difference between gross rainfall and net rainfall (throughfall + stemflow)^2^, which is however limited to specific locations and short periods. In situ-based studies reported substantial variations in *E*_*i*_ ranging from 10 to 50% of gross rainfall depending on vegetation attributes (plant functional types [PFT], leaf area index [LAI], etc.), rainfall regime characteristics, and evaporative demands^2,8–10^. While providing a first-order estimate of *E*_*i*_, such site-level measurements cannot scale up to continental or global scales since characteristics of drivers particularly rain events vary tremendously across space and time. Even within a site, this is still problematic because of horizontal and vertical variations in canopy characteristics or species. Global flux tower networks provide continuous eddy-covariance (EC) measurements of water and energy fluxes, and encompass a wide range of vegetation and meteorological conditions^11^. EC measurements do offer the opportunity to measure latent heat flux (LE, ET in the form of energy) at the ecosystem level as opposed to the tree level. Such ecosystem-level measurements have great capacity to extrapolate to large spatial scales by leveraging machine learning (ML) algorithms and Earth observations^12–15^. Nevertheless, EC towers do not directly measure *E*_*i*_, or indirectly partition the *E*_*i*_ part of this water flux.

Because of this critical data gap, global mapping of the *E*_*i*_ flux has generally used process- or physically based models^16–19^. Lacking mechanistic understanding of *E*_*i*_ development, the rainfall interception process is often overly simplified in current land-surface models (Table S1), many using an empirical relationship of *E*_*i*_ with gross rainfall and leaf area^20^, without explicit physical constraints of canopy energy and water budgets. One prominent physically based method is the Gash’s analytical model^17,21^. This model estimates *E*_*i*_ by additively calculating evaporation in the moistening, saturating and drying phases of rainfall interception during discrete events, which accounts for both canopy and rainfall characteristics^21^. However, this analytical model relies on several simplified assumptions that may not necessarily hold in real-world situations, for example, rain events are spaced sufficiently far apart such that canopy dries out completely^21^. To date, while extensive efforts has been devoted to refining *E*_*i*_ formulations in models^22,23^, these are, to a high degree, hindered by the lack of reliability and sparsity of observation-based *E*_*i*_ estimates at large scales for benchmarking model results.

The potential to partition *E*_*i*_ from LE measurements at EC flux towers is underexplored. Compared with leaf transpiration and soil evaporation, the *E*_*i*_ flux occurs exclusively under wet canopy conditions, and is a primary component of ET during or shortly after rain events^24^. Since *E*_*i*_ occur only for conditions of wet canopy, separating wet spells (during rainfall or the post-rainfall drying phase lasting 2–8 h^25^) from dry spells, offers a promising avenue for disentangling and estimating the *E*_*i*_ component from the total LE flux using EC measurements around the globe. A previous study^26^ developed an innovative empirical EC-based method for estimating *E*_*i*_, in which this *E*_*i*_ flux was determined as the excess evaporation occurring during and after rain events relative to baseline evaporation time series that are scaled from net radiation using an empirical relationship built during dry periods. This EC-based method was proven effective for estimating *E*_*i*_ at an old-growth rainforest in eastern Amazonia, showing that *E*_*i*_ accounted for 7.8–18% of rainfall during daytime rain events with a range of intensity^26^. Nevertheless, the generalization of this approach to other micrometeorological conditions and ecosystem types is not verified, and simple empirical models are insufficient to quantify the complexity of rainfall evaporation.

**Fig. 1:**
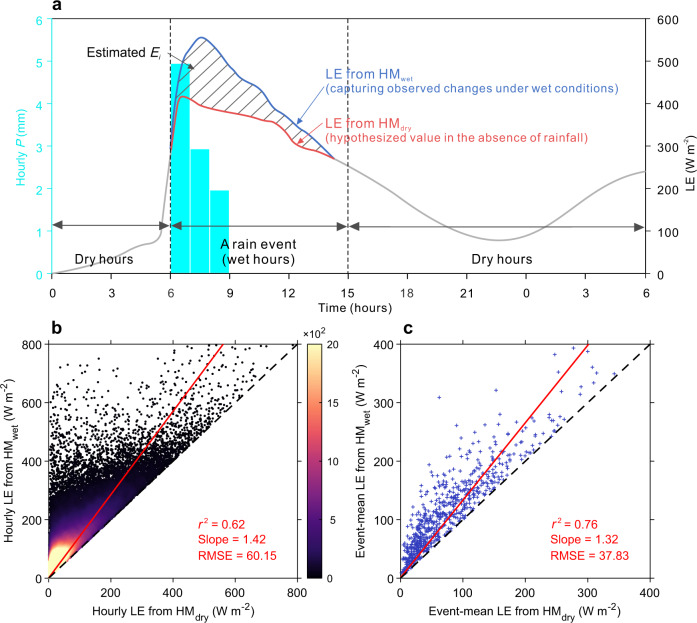
LE estimates by the hybrid models trained with wet samples (HM_wet_) versus dry samples (HM_dry_). **a** The conceptual framework to separate interception evaporation (*E*_*i*_) from tower-observed latent heat flux (LE). Cyan bars show hourly precipitation (*P*). The blue curve shows the response of LE simulated by HM_wet_ with accounting for rainfall occurrence, and red curve shows the hypothesized LE that would occur in the absence of rainfall based on HM_dry_. The dashed area represents the difference between the two LE estimates, which is used as an estimate of *E*_*i*_ flux during the event. **b** Scatterplots of LE estimated by HM_wet_ (*y* axis) against that by HM_dry_ (*x* axis) across all available site-hour samples. The color scale represents the density of samples (unitless). **c** Scatterplots of event-mean LE estimated by HM_wet_ against that by HM_dry_ across all rain events. In **b** and **c**, the red solid and black dash lines show the best-fit regression (intercept forced to be zero) line and identity line, respectively.

In this study, we modify the above EC-based method to be suitable for use worldwide, taking advantage of two hybrid models merging physics and machine learning (Methods). The hybrid models conserve energy at the land surface and use a resistor approach to estimate LE, which show better generalization to unseen (out-of-sample) conditions and non-linear processes (e.g., contrasting behaviors in dry versus wet seasons^27^), while also ensure physical consistency^28^. One model is trained with 146,608 wet samples from 29,985 rain events detected in the flux tower measurements (HM_wet_), and the other model is trained with 287,764 dry samples (HM_dry_) (“Methods”; Figs. S1 and S2), i.e., having not seen wet conditions when *E*_*i*_ occurs. The two models use a common set of environmental predictors including air temperature, net solar radiation (*R*_*n*_), wind speed, vapor pressure deficit (VPD), PFT and LAI (Fig. S1). The HM_wet_ uses an additional latent predictor called canopy water storage (CWS), which is inferred with a neural network from vegetation attributes and eight variables describing rainfall characteristics within the events (Methods; Fig. S1) in order to optimize the LE prediction in wet conditions. The model not having seen rainfall events (HM_dry_) provides the baseline LE estimate contributed by transpiration and soil evaporation (Fig. 1a). Hence, the difference between HM_wet_ and HM_dry_ naturally filters out the LE components of transpiration and soil evaporation, offering an indirect estimate for *E*_*i*_ (Fig. 1a). Importantly, the models allow for upscaling site-based *E*_*i*_ estimates to the global scale with the geo-spatial information of predictors available from Earth observations and climate reanalysis (“Methods”).

## Results

### LE estimates by HM_wet_ and HM_dry_

The hybrid models, established at hourly timescale, show a good performance in reproducing site-level LE observations under both wet and dry conditions (*r*^2^ > 0.75, RMSE < 60 mm) (Fig. S3). Given the natural variability of turbulence at EC towers, this validation indicates strong predictive power of the hybrid models to capture hourly LE dynamics. With all predictors (except for CWS) being consistent, the LE estimated by HM_wet_ is overall 42% greater than that by HM_dry_ at the hourly scale, and 32% greater at the event scale (Fig. 1b, c), demonstrating the important additional water flux during and shortly after rainfall. Hence, the difference between HM_wet_ and HM_dry_ reflects an instantaneous *E*_*i*_ response to the additional rainfall inputs. This difference scales nearly linearly with average LE (Fig. 1b, c), suggesting a larger *E*_*i*_ flux in wetter periods or warmer climates.

**Fig. 2:**
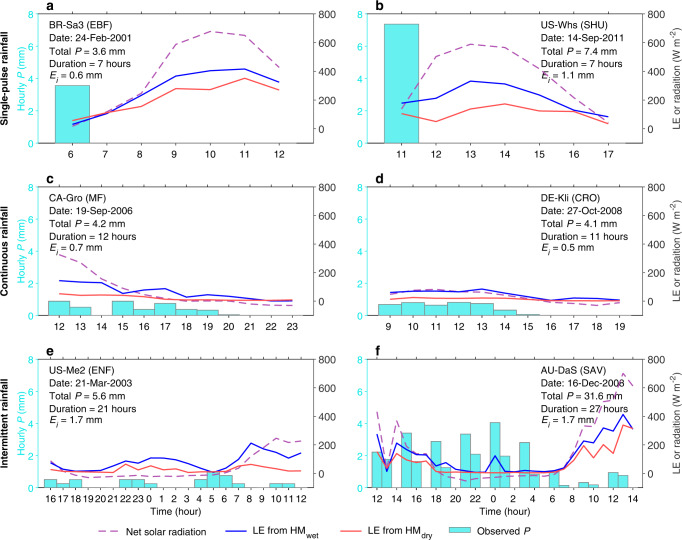
Cases of rainfall interception prediction in different rain events. Hourly time series of latent heat flux (LE) for several cases of rain events, as estimated by hybrid models trained with wet samples (HM_wet_, blue curves) and dry samples (HM_dry_, red curves). These cases are grouped into three general types: single-pulse rainfall (**a**, **b**), continuous rainfall (**c**, **d**) and intermittent rainfall (**e**, **f**). Cyan bars show the observed hourly precipitation (*P*), and purple curves show net solar radiation. X axis represents the local solar time from the start through the end of the event. Labels in the panels show the auxiliary information of the sites and rain events. EBF evergreen broadleaf forest, SHU shrubland, MF mixed forest, CRO cropland, ENF evergreen needleleaf forest, SAV savanna.

Our data-driven approach allows for assessing how *E*_*i*_ responds to rainfall occurrence on an hourly basis. We illustrate the model-predicted hourly time series for several cases of rain events grouped into three types: single-pulse rainfall, continuous rainfall and intermittent rainfall (Fig. 2). The result shows that *E*_*i*_ depends not only on the available water stored within the wet canopy, but also on the available energy to vaporize the water. During rainy hours, the evaporation rate of wet foliage is relatively small since rainfall often co-occurs with low incident solar radiation (Fig. 2). After the rainfall has ceased or during non-rain intervals, the evaporation of intercepted water follows increasing availability of solar radiation (energy-limited), and then decreases after the canopy has been gradually dried out (water-limited) (Fig. 2). The maximum evaporation rates occur under conditions with both abundant canopy water and also sufficient energy, often lagging behind the latest rainfall pulse for a few hours (Fig. 2). We emphasize that this expected physical behavior is not imposed in the model but learned from observations, further building confidence in the mechanistic representation of the hybrid models.

### The fraction of rainfall partitioned to *E*_*i*_ and its drivers

**Fig. 3:**
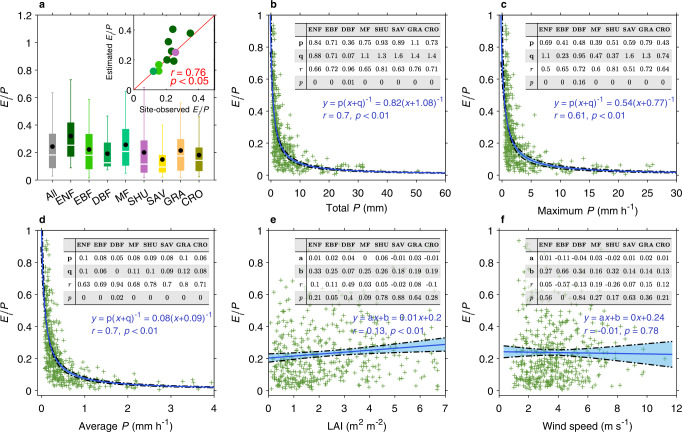
Event-level fraction of rainfall interception loss (*E*_*i*_/*P*) and its driving factors. **a** Median (white line), average (black dots), interquartile range (shading), and the 5th and 95th percentiles (whiskers) of *E*_*i*_/*P* across eight plant functional types (PFTs). The upper right scatterplot in **a** compares the estimated *E*_*i*_/*P* with observed *E*_*i*_/*P* at geographically close sites (Methods), with colors denoting the corresponding PFT defined by the bars. The identity line is shown in red. **b**-**f** Scatterplots of the event-mean *E*_*i*_/*P* ratio against four potential driving factors including total precipitation (*P*) (**b**), maximum hourly *P* (**c**), average hourly *P* (**d**), leaf area index (LAI) (**e**) and wind speed (**f**). The relationship is characterized for both all available rain events (dots), and for the events grouped into each of the eight PFTs (inset tables). The relationships with three metrics of rainfall characteristics are fitted with a rational model, while the others are fitted with a linear model. Quantities p, q, a and b are regression coefficients. Blue curves represent the best-fitted regression, with the shading showing the 95% confidence intervals. EBF evergreen broadleaf forest, DBF deciduous broadleaf forest, ENF evergreen needleleaf forest, MF mixed forest, SAV savanna, SHU shrubland, GRA grassland, CRO cropland.

The fraction of *E*_*i*_ in gross precipitation (*E*_*i*_/*P*), i.e., the incoming precipitation at the top of the canopy, measures the relative importance of *E*_*i*_ in surface water budget. Our result shows that the *E*_*i*_/*P* varies tremendously from near 0% to 100% (Fig. 3), across rain events, with a median of 18.3% and a mean of 24.4% (Fig. 3a). The event-level estimate is quantitatively comparable to the global *E*_*i*_/*P* estimate (median: 21.8%; mean: 25.0%) reported by a recent meta-analysis of field studies^10^. Among the four forest biomes, the highest *E*_*i*_/*P* values are identified for evergreen needleleaf forests (32.0%, mean only), followed by mixed forests (25.6%) and evergreen broadleaf forests (22.2%), while the lowest values are detected for deciduous broadleaf forests (19.2%) (Fig. 3a). Non-forest biomes (often in relatively dry regions) show relatively lower *E*_*i*_/*P* values (shrub: 20.1%, grasslands: 21.8%, savanna: 20.1%) than forest biomes. By comparing our EC-based *E*_*i*_/*P* values against a set of geographically close in situ observations (Methods), we find a general agreement across available sites (*r* = 0.76, *p* < 0.05) (Fig. 3a). This validation against independent ground measurements confirms the capacity of our hybrid models for inferring *E*_*i*_ variations across biomes.

To understand the determinants of *E*_*i*_/*P* variations, we analyze the relationship between *E*_*i*_/*P* and its drivers across all available events, for individual PFTs (Fig. 3). We find that rainfall characteristics (including total rainfall amount, maximum hourly rainfall and average hourly rainfall; Fig. 3a–c), rather than vegetation attributes (Fig. 3e), play a dominant role in determining *E*_*i*_/*P* variations. The *E*_*i*_/*P* ratio is inversely related to average and maximum raining rates, indicating that more intense rain events cause less rainfall partitioned into *E*_*i*_ (Fig. 3a–c). About 3/4th of rain events that have average rainfall rate greater than 1.0 mm h^−1^ show a *E*_*i*_/*P* ratio lower than 5% (Fig. 3c). This finding is in line with field observations that canopy has greater potential to intercept rainfall in drizzle and light rain conditions than during short rainstorms^29,30^. During heavy rain events, interception first increases proportionally with rainfall until the canopy water storage reaches its saturation level as the area coverage of the water on the leaves or stems is very high and the surface tension cannot hold any more water droplets on the plants^29,30^. After reaching the maximum interception capacity, the additional rainfall becomes throughfall or stemflow and will no longer contribute to *E*_*i*_.

*E*_*i*_/*P* is positively correlated to LAI across all available events (*r* = 0.13, *p* < 0.05) as denser canopies can intercept more water, yet this relationship is weak and insignificant for most PFTs (Fig. 3e). This weak *E*_*i*_/*P* dependence on LAI is confounded by the overwhelming effect of rainfall intensity. For a specific LAI level, there exists a tremendous variability in the types of rainfall events, encompassing a wide range of intensity (Fig. S4) and dampening potential *E*_*i*_ response to LAI. The *E*_*i*_ capacity also depends on other plant characteristics such as leaf morphology, leaf inclination, leaf texture, canopy architecture and the overstory-substrate structure^31,32^. These factors are to some extent captured by PFT and LAI, but are hard to infer in the absence of available measurements. Similarly, a weak relationship between *E*_*i*_/*P* and wind speed can be found due to the confounding effects of the diverse rainfall regimes. A significant negative correlation between *E*_*i*_/*P* and wind speed (*r* = −0.57, *p* < 0.01) is however detected for evergreen broadleaf forest (Fig. 3f). Wind speed affects *E*_*i*_ through two counteracting processes: wind blows over raindrops into the canopy interior and promotes evaporation loss of wet leaves, while precluding water collection by enhancing air motion and swaying canopy leaves^33^. This negative relationship for broadleaf species suggests the second mechanism plays a dominant role.

### Global and zonal contribution of *E*_*i*_ to gross rainfall

**Fig. 4:**
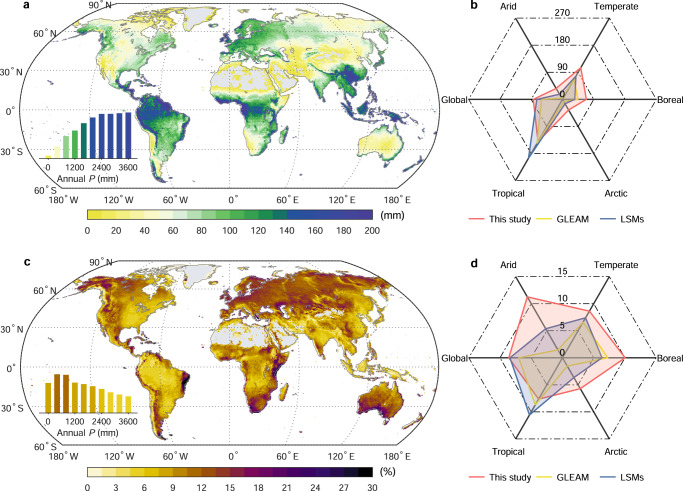
Spatial variations in annual mean intercepted rainfall (*E*_*i*_) and its fraction of total rainfall (*E*_*i*_/*P*). Spatial patterns represent multi-year annual mean *E*_*i*_ (**a**) and *E*_*i*_/*P* (**c**) during 2000–2020, with the inset histogram showing the mean value across precipitation (*P*) gradients. The radar plots on the right compare the *E*_*i*_ (**b**) and *E*_*i*_/*P* (**d**) values among three products and five climate zones (Fig. S2). GLEAM uses the Gash’s analytical model for *E*_*i*_ prediction. LSMs show the ensemble mean value of six land-surface models (“Methods”).

Using spatially explicit climatic and vegetation states as inputs, we produce an upscaled global data-driven estimate for cumulative rainfall interception over 2000–2020 (“Methods”). Averaged over the entire period, the annual accumulated global *E*_*i*_ is 84.1 ± 1.8 (mean ± 1-SD, based on interannual variability) mm, which accounts for about 8.6 ± 0.2% of total incoming rainfall. The spatial pattern of mean *E*_*i*_ shows strong similarity to that of precipitation and LAI climatology, as expected, with the highest *E*_*i*_ occurring in the wettest and most densely vegetated tropical regions (Fig. 4a, b). Importantly, *E*_*i*_ does not increase proportionally with precipitation across global lands. Strikingly, the *E*_*i*_/*P* ratio tends to increase with rainfall amount in relatively dry regions (*P* < 800 mm yr^−1^), which however shifts to decrease in humid regions (*P* > 800 mm yr^−1^) (Fig. 4c). The contrasting behavior of *E*_*i*_/*P* across moisture gradients is due to tradeoff between effects of vegetation cover and rainfall characteristics. In dry regions with low-rainfall intensity, the *E*_*i*_/*P* increases with vegetation cover. However, in humid regions particularly tropics dominated by short-duration convective rainfall events, the fraction of annual rainfall contributed by heavily raining hours (>1.0 mm) and the average rainfall rates are both much greater than that in dry regions (Fig. S5), favoring lower *E*_*i*_/*P*, as demonstrated by our site-based analyses (Fig. 3c, d).

We further compare our data-driven results with available global *E*_*i*_ estimates from GLEAM (which uses the Gash’s analytical model) and state-of-the-art land-surface models (LSMs, Table S1). Globally, our data-driven approach produces greater annual mean *E*_*i*_ than estimates from GLEAM (60.2 mm) and LSMs (73.9 mm; ranging from 18.9 mm in CABLE-POP to 100.3 mm in ISAM), as well as the *E*_*i*_/*P* (GLEAM: 7.0%, LSMs: 8.4% [2.2–13.0%]) (Fig. 4b, d). By categorizing global lands into five dominant climate zones (tropical, arid, temperate, boreal, polar), we show that all products have similar spatial structure of *E*_*i*_ despite having varying values. However, both GLEAM and LSMs fail to reproduce the spatial structure of the *E*_*i*_/*P* ratio, with much higher *E*_*i*_/*P* in tropical wet climates (this work: 7.6%, GLEAM: 8.7%, LSMs: 10.6%) than in arid (this work: 11.3%, GLEAM: 1.5%, LSMs: 5.4%) or boreal climates (this work: 10.0%, GLEAM: 7.3%, LSMs: 6.3%) (Fig. 4d). This systematic bias is likely caused by the fact that, in empirical physically based models, current parameterization of the *E*_*i*_ process overly relies on leaf area or canopy cover, and correspondingly, underestimates the dominant role of rainfall intensity in determining rainfall partitioning to *E*_*i*_ especially in humid regions (Fig. S6). In addition, the large inter-model spread of *E*_*i*_ (and *E*_*i*_/*P*) estimates (Fig. S7) highlights the need for observational constraint on this flux and further improvement of process representation.

### Rainfall regime characteristics drive recent *E*_*i*_ changes

**Fig. 5:**
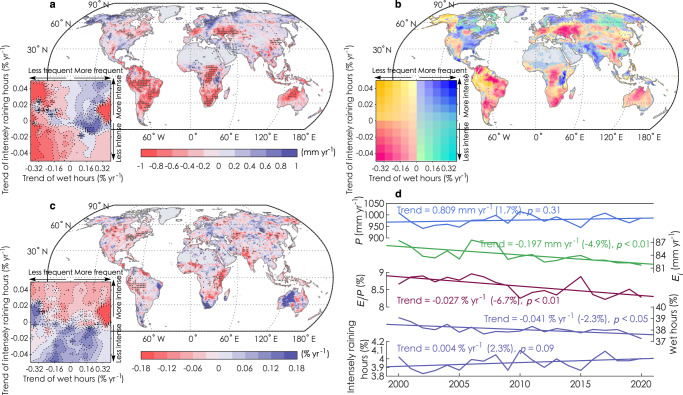
Temporal changes of rainfall interception loss (*E*_*i*_) for 2000–2020 controlled by rainfall (*P*) characteristics. Spatial patterns of the linear trend in annual *E*_*i*_ (**a**), rainfall frequency and intensity (**b**) and the ratio of *E*_*i*_ to *P* (*E*_*i*_/*P*) (**c**), all calculated for the 2000–2020 period. Stipples show statistically significant (*p* < 0.05) trends. The inset plots at the lower left corners of **a** and **c** show the distribution of the value in the space of rainfall frequency and intensity changes. Rainfall frequency and intensity use the fraction of wet hours and intensely raining hours as indicators, respectively. **d** Trajectories of global mean *P*, *E*_*i*_, *E*_*i*_/*P*, rainfall frequency and intensity during 2000–2020. Labels alongside the best-fitting lines indicate the linear trends, significance, and the overall changes relative to its mean value during 2000–2020 (in brackets).

We next assess how the global *E*_*i*_ flux has changed over the last two decades (Fig. 5). During 2000–2020, global mean annual precipitation has remained almost unchanged with an insignificant trend of +0.81 mm yr^−1^ (*p* > 0.1) (Fig. 5d). Our data-based approach however estimates that global mean annual *E*_*i*_ has significantly decreased by 4.9% (percent change relative to the climatology; average rate: −0.20 mm yr^−1^, *p* < 0.01), which leads to a global decline in the *E*_*i*_/*P* ratio by 6.7% (*p* < 0.01) (Fig. 5d). Given the dominant effect of rainfall characteristics on *E*_*i*_/*P* (Fig. 3a–c), we examine concurrent changes in rainfall frequency (*F*_rain_) and intensity (*I*_rain_), using the fraction of wet hours (0 mm h^−1^ < *P* < 90^th^ percentile) and intensely raining hours (> 90th percentile of multi-year rainy hours) as indicators, respectively (Methods). We diagnose a decreasing fraction of wet hours (−2.3%, *p* < 0.01) and an increasing fraction of intensely raining hours (+2.3%, *p* = 0.09) during 2000–2020 (Fig. 5d). These trends suggest that global land rain regimes have shifted to be less frequent and more intense, both becoming less favorable for *E*_*i*_ generation. Globally, the year-to-year variations of *E*_*i*_ are significantly and positively corrected with *F*_rain_ (Pearson correlation: *r* = 0.55, *p* < 0.05) but not with *I*_rain_ (*r* = 0.14, *p* > 0.1) (Fig. S8a, b). Oppositely, the *E*_*i*_/*P* is significantly and negatively correlated with *I*_rain_ (*r* = −0.61, *p* < 0.05) but not with *F*_rain_ (*r* = 0.28, *p* > 0.1) (Fig. S8c, d). This indicates that rainfall frequency alters average *E*_*i*_, while rainfall intensity determines rainfall partitioning between *E*_*i*_ and other fluxes.

The changes in rainfall and its distribution characteristics are spatially diverse (Fig. 5b), leading to a strong heterogeneity in *E*_*i*_ changes (Fig. 5a). *E*_*i*_ shows a decreasing trend over 74.2% of tropical lands and 57.5% of extratropical lands, with the few increasing areas identified over the eastern U.S., Middle East and northern high latitudes. By summarizing the *E*_*i*_ trend in terms of *F*_rain_ and *I*_rain_ changes (areas with significant *P* trend are excluded), we confirm that *F*_rain_ is the most important factor, compared to *I*_rain_, to explain the spatial variations of *E*_*i*_ trend (Fig. 5a). Most global land areas that see a *E*_*i*_ decline corresponds to regions with less frequent rainfall, such as the vast tropical regions (Fig. 5a, b). The trend of *E*_*i*_/*P* presents even stronger spatial heterogeneity, with 60.3% of global lands being negative and the remaining 29.7% being positive (Fig. 5c). In contrast to the *E*_*i*_ trend, yet in concert with the global result, the spatial variations of *E*_*i*_/*P* trend are modulated to a greater extent by *I*_rain_ than *F*_rain_ changes (Fig. 5c). The *E*_*i*_/*P* ratio decreases for land areas where precipitation gets more intense, such as the Amazon rainforest, eastern Africa and India, and correspondingly, this ratio tends to increase where precipitation is less intense, such as in western Australia and eastern Europe (Fig. 5b, c).

## Discussion

By integrating EC measurements, meteorological and reanalysis observations in a hybrid data-driven approach, we generate the data-driven and spatially explicit estimate of global rainfall interception over the past two decades (2000–2020), and characterize its overall contribution to the global water balance. We estimate that *E*_*i*_ accounts for around 8.6% of gross land rainfall, but can be higher than 15% in some low-rainfall-intensity areas. Rainfall interception is usually regarded as a non-beneficial water use, as this flux does not benefit plant productivity or societal water needs^34^. A knowledge of this flux, however, has implications for water-oriented forest planning and management especially in arid regions^35,36^. For instance, in regions undergoing massive forest loss (agricultural clearing, logging, diebacks from fires or droughts), the *E*_*i*_ loss would almost completely convert into extra water inputs to the catchment water cycle. In an opposite case, for regions undergoing large-scale afforestation practices, the rainfall interception adds to the concomitant loss of transpiration^37^. This hydrological process could be a significant driver of observed decline in available surface water over intensely afforested areas, yet has not been accurately assessed or event accounted for^38^. We thus call for consideration of this additional water loss by interception when formulating the impact of future afforestation programs initialized to combat climate change.

Our study demonstrates the capacity of hybrid modeling for capturing *E*_*i*_ dynamics across climates/biomes, under the assumption that relationships built over dry periods can be applied to wet periods. However, we are aware of potential biases in the *E*_*i*_ estimate in certain circumstances where the assumption might not hold true. For example, during rainfall, the intercepted water could reduce transpiration by covering some stomata openings and limiting leaf-air water vapor exchanges^39,40^, which would translate to a negaitve bias in the *E*_*i*_ estimate. This bias is alleviated by the fact that most plants stomata are located on the lower side of the leaves, and thus do not direclty interact with the intercepted rainfall unless during storms. Moreover, even though HM_dry_ exposed only to dry conditions doesn’t encapsulate this direct physical constraint of water, it still has adaptive capacity to wet periods because: (1) it has explicitly accounted for leaf energy balance; (2) it has captured other meteorological factors accompanying rainfall occurrence (e.g., high humidity and low radiation) that constrain leaf transpiration. Potential biases from soil evaporation and transpiration fluxes should also be noted in water-limited biomes. In such areas, our estimated *E*_*i*_ is to some degree confounded by soil evaporation and transpiration pulses right after rainfall when there are both abundant soil water and still relatively high atmospheric demand for water^41^. Another source of uncertainty is that ML algorithms targeting at predicting fine-scale targets (e.g., hourly *E*_*i*_) have inherent deficiency at capturing impacts of slowly evolving factors (e.g., LAI). Although leaf area is experimentally identified as a key driver of LE and *E*_*i*_ variations^8,10,42^, this effect is however of secondary importance under our hybrid framework (Fig. 3e, Fig. S9). Future research needs to better encapsulate the longer-term effect of ongoing vegetation changes^37^ on *E*_*i*_ dynamics, particularly for more targeted assessments of vegetation impact on regional water resources.

Our data-driven results suggest that rainfall characteristics are the primary driver of rainfall interception capacity at large spatial scales. This is supported by several experiment-based studies^9,43^, but is contrary to some others since in situ plots are often situated close to each other and observe similar rainfall patterns. The critical role of rainfall characteristics challenges the widely adopted linear scaling of *E*_*i*_ with rainfall in models based on LAI or canopy cover fraction^20^, and also highlights that insufficient representation of sub-daily rainfall variability (e.g., prediction on a daily or longer basis^44^) would yield a substantial bias. We demonstrate a reduced partitioning of incoming rainfall into interception loss in less frequent and more intense rain events under climate change. This dependence has produced a significant decreasing trend of *E*_*i*_ since 2000^45^, and will likely determine future *E*_*i*_ changes in response to shifts in rainfall characteristics. In the warming future, state-of-the-art climate models project a robust intensification of rainfall extremes and decrease of rainfall frequency globally^46–48^. Hence, the present decreasing trend of interception loss should continue in future, thus increasing soil moisture and runoff. This altered rainfall partitioning could have two counteracting effects that require further in-depth investigation: (1) supplying water needed for ecosystem functions and human activities; (2) increasing flood risks as the extra water occurs mainly during intense rainfall events.

## Methods

### FLUXNET measurements

Global collection of eddy-covariance (EC) measurements at flux towers were obtained from the FLUXNET2015 Tier 2 database^11^, which contains gap-filled, half-hourly measurements of carbon, water and energy fluxes, and meteorology. We here used measurements of latent heat flux (LE), sensible heat flux (*H*), air temperature (*T*_*a*_), precipitation (*P*), vapor pressure deficit (VPD), net solar radiation (*R*_*n*_), ground heat flux (*G*), wind speed (WS), as well as auxiliary information of site locations, plant functional types (PFTs), canopy and tower heights. The data originally consist of 212 sites that encompass 13 PFTs defined by the International Geosphere Biosphere Programme (IGBP).

The following data filtering was applied to the half-hourly flux data: (1) sites (or periods) without sufficient measurements required for the hybrid model, as listed above, were excluded; (2) half-hourly data with negative LE or labeled as having ‘poor quality’ were excluded; (3) in situ measurements generally have small footprints, thus sites where the site-level PFT is not representative of the dominant PFT retrieved from satellite grids were filtered out. Here, to ensure site availability in arid regions, woody savanna (WSA) was combined into savanna (SAV), open shrublands (OSH) and closed shrublands (CSH) were combined into shrublands (SHU); (4) measurements with *T*_*a*_ < 0 °C were excluded, to avoid confounding effects of snowfall interception. The data screening led to a subset of 76 sites that encompasses eight major vegetation types: EBF (evergreen broadleaf forest), DBF (deciduous broadleaf forest), ENF (evergreen needleleaf forest), MF (mixed forest), SAV, SHU, GRA (grassland) and CRO (cropland) (Fig. S2; Table S2). Among these, 48 use the open-path (OP) EC system and the rest use the closed-path (CP) EC system (Table S2), which differ in deployment and data post-processing measurements and thus the ET estimates^49^. Site-level LAI time series were not directly available, so this information was extracted from satellite-retrieved 8-day LAI maps based on site locations (details of LAI and PFT data in “Global *E*_*i*_ mapping driven by satellite and reanalysis data”). To alleviate the influence of non-vegetated surfaces within the grids, for each site, the extracted grid-mean LAI was multiplied by the ratio of site maximum LAI to grid maximum LAI. Maximum LAI for 39 flux sites was collected by literature review^50^, and the scaling factor for the remaining sites were filled with the nearest site of the same PFT.

We aggregated all half-hourly time series of all variables to hourly, to accommodate the hourly reanalysis data used for global mapping of *E*_*i*_. Previous studies demonstrated a systematic underestimation of LE measurements during or shortly after rainfall, because the low-pass filtering of water vapor is inherently flawed under raining and high-humidity conditions^51,52^. Assuming a dependence of the latent energy ratio (LER, defined as LE/(*R*_*n*_ − *G* − *H*)) on relative humidity (RH) and rainfall intensity, we applied a neural network (NN) to correct for potential biases in LE following a recent study^47^. Specifically, we first built for each flux site a NN to model LER as a function of RH and log-transformed hourly *P*. Using the LER predictions driven by observed predictors (LER_pred_), we next corrected hourly LE to ensure that LER was set at the reference level with moderate RH (50%) and no rain (*P* = 0 mm h^−1^), expressed as:

1 $${{{\rm{LE}}}}_{{{\rm{cor}}}}={{{\rm{LE}}}} \times {{{\rm{LER}}}}_{{{{\rm{pred}}}}[{{{\rm{RH}}}}= 50 \%,\, P=0]}/{{{\rm{LER}}}}_{{{\rm{pred}}}}$$

where LE_cor_ is the LE flux after correction for RH and *P* dependence, LER_pred[RH=50%, *P*=0]_ is the reference LER corresponding to moderate RH and no rain. Last, a Bowen ratio method^49^ was applied to correct for potential incomplete energy balance closure remained in the hourly LE data^53^.

### Splitting wet and dry hours

For each flux site, we separated the hourly time series into wet and dry hours, as inputs to HM_wet_ and HM_dry_ (details below), respectively. Wet hours were defined as those within a rain event. Rain events were identified with site-observed *P* time series based on the following three principles: (1) it starts with hourly *P* ≥ 0.5 mm; (2) the following 6 hours (12 h if occurred during nighttime) after the event ceases were also included in the same event, because evaporation of wet foliage often lags behind rain occurrence. If there was an hour with *P* ≥ 0.5 mm during the following 6 h, this event was extended to another 6 h until none in the following 6 h exceeded this threshold. (3) If an event lasted longer than 60 h, the whole rainfall period was split into several shorter rain events that started with *P* ≥ 1 mm and spaced longer than 6 hours. Based on this definition, an event lasts a couple of hours, and may contain a few non-rain (*P* = 0 mm) intervals (Fig. S1b). Those consecutive rainy hours (*P* > 0 mm) split by non-rain intervals were considered as individual rainfall pulses (Fig. S1b). Last, those non-rain hours outside the rain events were defined as dry hours. In total, we obtained 146,608 wet samples from 29,985 individual rain events, as well as 287,764 dry samples.

### Hybrid model architecture, training, and prediction

The hybrid model for quantifying LE is a physics-constrained machine learning (ML) model developed by Zhao et al.^28^. This model integrates traditional ML model with a physically based model, thus leveraging the strengths of ML (strong predictive ability and data adaptiveness)^54,55^ and physical modeling (theoretical foundations, interpretability and extrapolation capacity)^28,56^. In this model, the physical part of the model retrieves surface resistance (*R*_*s*_) from LE by inversing a quadratic Penman-Monteith (PM) equation, and the ML part predicts the logarithm value of *R*_*s*_ (which is more normally distributed than *R*_*s*_), as a set of environmental factors (Fig. S1a). A quadratic PM equation is used in the loss function to ensure that the LE predictions conserve the surface energy balance and meet the physical constraint that ET is a turbulent diffusion process driven by vapor pressure gradients^28^. The ML algorithm used in the model is a feedforward NN. Our model setup of NN was consistent with what used in Zhao et al.^28^. The hybrid model has proved to outperform pure NN in ET prediction particularly under climate extremes and for out-of-sample extrapolation^15,28^. This improved behavior is the rationale for the use of this approach such that a hybrid model fitted outside of rainy conditions (HM_dry_) can be compared to the hybrid model fitted during and right after rainy conditions (HM_wet_).

The workflow of model training and prediction is shown in Fig. S1. We first built a hybrid model (i.e., HM_dry_) using dry samples of LE and its predictors including *T*_*a*_, VPD, *R*_*n*_, WS, LAI and PFT. Before the model training, all input variables (except PFT that used the original category values) were normalized by the mean and standard deviation (SD) to have zero mean and normalized variance. We next fed the HM_dry_ with its predictors observed under wet conditions, which estimated the hypothesized LE values without canopy interception (Fig. 1a), as this model only saw dry conditions where rainfall interception was absent during its training. The wet samples provided LE observations that captured the contribution of rainfall intercepted by canopies. Thus, the difference between observed LE and HM_dry_-predicted LE was used as a proxy for the amount of vaporized canopy water storage (CWS) from interception. Note that this parameter also encapsulates energy availability for evaporation at hourly scales (Fig. S1b), so this can also be interpreted as canopy interception capacity, which accounts for both water and energy constraints. We then built a NN to predict the CWS (as a latent variable in HM_wet_) since this parameter is not directly observable but is necessary for the global mapping of *E*_*i*_ using climate reanalysis. Predictors used here were vegetation states (LAI, PFT) and eight variables describing rainfall characteristics including: (1) accumulated *P* since the start of the event; (2) average hourly *P* since the start of the event; (3) maximum hourly *P* since the start of the event; (4) *P* of the current hour; (5) accumulated *P* of the last rain pulse; (6) maximum hourly *P* of the last rain pulse; (7) number of non-rain hours since the last rain pulse; (8) end timing of the last rain pulse. Further, we built HM_wet_ in a similar manner as HM_dry_, except using wet samples and using CWS as an additional predictor. Last, the difference between LE estimates by HM_wet_ and HM_dry_, forced identically by wet samples, was regarded as an estimate for the *E*_*i*_ flux. A small fraction of estimated CWS and *E*_*i*_ values were negative (<0 mm) due to inherent noise in the flux tower measurements. These anomalous values were removed when reconstructing the predicted time series for individual rain events.

### Validation against in situ *E*_*i*_ measurements

To validate our EC-based *E*_*i*_ estimates, we obtained 981 in situ observations of *E*_*i*_ from a recent meta-analysis paper^10^ for comparison. Such ground-based *E*_*i*_ was often observed indirectly as the difference between gross rainfall measured above canopy or at a neighboring open land, and the sum of the throughfall and stemflow sampled simultaneously on the forest floor. This site-level validation was subject to issues of limited spatial representation and mismatch of measuring periods, particularly given the spatially and temporally varying rainfall inputs that strongly affect *E*_*i*_. Thus, this validation was based on the *E*_*i*_/*P* ratio, rather than *E*_*i*_, to ensure better comparability. We selected sites that met the two criteria: (1) both individual- and community-level data were provided, but only community-level measurements were used to match our ecosystem-level EC-based estimates; (2) for each flux site, we only selected in situ site(s) of the same PFT and located within 500 km of the target site. Applying the criteria led to a small set of 10 in situ observation sites that encompassed four vegetation types (ENF, EBF, DBF and SHU) (Fig. 3a). In this comparison, the *E*_*i*_/*P* at flux sites was averaged over all detected rainfall events, and that of in situ sites was also averaged over plots, rainfall events, and/or repeated experiments.

### Global *E*_*i*_ mapping driven by satellite and reanalysis data

We used the well-trained hybrid models to upscale site-inferred *E*_*i*_ from towers to the global scale using globally available predictor variables from climate reanalysis and satellite observations. Gridded hourly meteorological variables required as model inputs (*T*_*a*_, VPD, *R*_*n*_, WS, *P*, *G*) were derived from the ERA5 climate reanalysis data^57^. This product is available at 0.25° × 0.25° grids since 1979 and has shown high consistency with observations^57^. Global LAI maps were derived from the MOD15A2H (C6) product, available as 8-day composites with 500-m spatial resolution since 2000. Global IGBP PFT maps were from the MCD12C1 (C6) product, available at 0.05° × 0.05° grids since 2000. We predicted *E*_*i*_ for the overlapping 2000–2020 period, and at 0.5° × 0.5° global grid after regridding all forcing data to this common spatial resolution. We identified the dominant PFT with the largest percent cover after aggregating the percent fraction of each land cover to 0.5° × 0.5° grid. LAI at 8-day intervals was interpolated to hourly using a cubic smoothing spline algorithm. Unlike the use of site-corrected LAI for model training, global prediction of *E*_*i*_ instead used the original LAI values.

First, we extracted predictor variables for hours and grid cells that were within a rain event (based on the definition in “Splitting wet and dry hours”) by searching the *P* time series over the past 60 h. Second, CWS was approximated for each wet hour and grid using LAI, PFT and the eight variables of rainfall characteristics. Third, all grid-based variables (except CWS) were normalized by the mean and SD of site-based values, with additional verification that they generally fell within the range of site-based variables (not shown). The site-based CWS and grid-based CWS were quantitatively different, which could bring a systematic bias in *E*_*i*_ estimates since only the HM_wet_ has incorporated this parameter. Therefore, global grids of CWS were normalized using the mean and SD calculated for those grids surrounding the site locations (within a 5° × 5° window), instead of that from site-based values. This ensured that normalized CWS values from sites and surrounding grids were quantitatively similar. Fourth, we re-ran the hybrid models to predict *E*_*i*_ for wet hours/grids driven by the predictors, and reconstructed the global *E*_*i*_ maps. Finally, the predicted gridded *E*_*i*_ was multiplied by the fraction of vegetated cover (derived from the MOD44B product) to exclude contributions from non-vegetated surfaces within the grids.

Previous model-based studies highlighted the effect of sub-grid rainfall variability on rainfall interception^58,59^. There was a sizable fraction of rainfall at coarse grids contributed by hourly *P* < 0.5 mm. The light rain excluded from rain events also contributed to *E*_*i*_ due to the strong spatial heterogeneity of rainfall within the grid. We here adopted the following method to solve this problem. We assumed the *E*_*i*_/*P* ratio increases linearly with smaller rainfall amounts for light rain (*P* < 0.5 mm) (Fig. S11). If the rainfall amount converged to zero, we expected that rainfall over canopy could be completely intercepted, hence the grid-mean *E*_*i*_/*P* ratio was approximated as the fraction of vegetated surface. When the rainfall amount was close to 0.6 mm (0.5–0.7 mm to ensure enough samples), we obtained the average *E*_*i*_/*P* ratio from the estimates by hybrid models for each PFT. As a result, we linearly interpolated the *E*_*i*_/*P* for any given PFT and rainfall amount between 0 to 0.5 mm, and used this ratio to calculate *E*_*i*_ in response to light rain (*P* < 0.5 mm outside rain events) (Fig. S11).

When attributing global and regional *E*_*i*_ (*E*_*i*_/*P*) changes, we introduced two measures of rainfall characteristics. Rainfall intensity was measured by the yearly fraction of intensely raining hours. Specifically, we determined for each grid the 90th percentile of hourly rainfall amounts across all rainy hours (*P* > 0 mm) during 2000–2020, and defined intensely raining hours as those with simultaneous rainfall exceeding the local 90^th^ percentile. Rainfall frequency was then measured by the yearly fraction of wet hours excluding those intensely rainy ones (0 mm <*P* < local 90th percentile).

### Process- and physically based *E*_*i*_ simulations

Available global *E*_*i*_ estimates from land-surface models (LSMs) and the Gash’s analytical model were used for comparison. We used an ensemble of six LSMs from the TRENDY (trends in net land–atmosphere carbon exchange) v7 project^60,61^, which provided *E*_*i*_ outputs for 2000–2018. These models included CABLE-POP, CLM5.0, ISAM, LPJGUESS, ORCHIDEE-CNP and SURFEX. For all LSMs, we used the simulation (S2) forced by varying both atmospheric CO_2_ and climate. We also obtained the *E*_*i*_ data from the Global Land Evaporation Amsterdam Model (GLEAM) v3.4a, in which the *E*_*i*_ estimation relied on the Gash’s analytical model^19^. This product is available at 0.25° × 0.25° global grids for 1981–2018. All model data were regridded to 0.5° × 0.5° spatial resolution for comparability. Note that, *E*_*i*_ estimation closely depends on the precipitation inputs, so the derivation of *E*_*i*_/*P* used the original precipitation forcing data, that is, CRU-NCEP for LSMs and MSWEP for GLEAM^19^. This comparison was also conducted for the five major global eco-climatic zones (tropical, dry, temperate, boreal and polar) according to the Köppen–Geiger climate classification (types A, B, C, D, E, respectively; Fig. S2).

## Supplementary information

Supplementary Information

## Data Availability

All observation and model data that support the findings of this study are available as follows. The FLUXNET2015 EC measurements are available at https://fluxnet.fluxdata.org/2015/12/31/fluxnet2015-dataset-release/; The ERA5 Reanalysis products are available at https://cds.climate.copernicus.eu/cdsapp#!/dataset/reanalysis-era5-single-levels?tab=form; The GLEAM v3.5b datasets are available at https://www.gleam.eu/; The MODIS LAI data are available at https://e4ftl01.cr.usgs.gov/MOLT/MOD15A2H.006/; The MODIS land cover maps are available at https://e4ftl01.cr.usgs.gov/MOTA/MCD12C1.006/; The Köeppen-geiger climate classification map is available at: http://koeppen-geiger.vu-wien.ac.at/prese nt.htm; The processed *E*_*i*_ data are available at: 10.5281/zenodo.7309030.

## Code availability

The processing MATLAB and Python codes are available from the corresponding author upon request.
