# Supplementary material for: Recent global decline in rainfall interception loss due to altered rainfall regimes
Source: Nat Commun. 2022 Dec 10;13:7642. doi: 10.1038/s41467-022-35414-y (PMC9741630; doi:10.1038/s41467-022-35414-y)
Supplement: Supplementary file 1 — Supplementary Information [file 41467_2022_35414_MOESM1_ESM.pdf]

Supplementary Information for

Recent global decline in rainfall interception loss due to altered rainfall regimes

by Xu Lian et al.

This PDF file contains:

Supplementary Tables 1-2

Supplementary Figures 1-11

Supplementary Table 1. Canopy interception schemes in TRENDY land surface models.

Commonly used abbreviations: *P* precipitation, *L* leaf area index, f_v, vegetative fractional cover, NA not available.

Model name	Model name abbreviation	E_i parameterization
Community Atmosphere Biosphere Land Exchange - Populations-Order-Physiology	CABLE-POP	Determined by L and canst1<br>*canst1*: max intercepted water by canopy
Community Land Model version 5.0	CLM5.0	$E_i = P\alpha \tanh(L+S)$<br>*L*, *S*: exposed leaf and stem area;<br>α: the fractional area of a leaf that collects water.
Integrated Science Assessment Model	ISAM	NA
Lund-Potsdam-Jena General Ecosystem Simulator	LPJGUESS	$I_{sc} = PLif_v$<br>$E_i = E_q \alpha w;\ w = \min\,(I_{sc}/[E_q\alpha], 1)$<br>$I_{sc}$: canopy interception storage capacity dependent on P, L, rainfall regime storage coefficients (*i*) and vegetative fractional cover (f_v); E_q: potentially evaporated water determined via the Priestley-Taylor method (using a maximum coefficient (α) of 1);<br>w: daytime canopy wetness duration.
Organising Carbon and Hydrology In Dynamic Ecosystems-CNP	ORCHIDEE-CNP	$E_i = \beta_i \rho (q_{sat} - q_a)/r_a$<br>$\beta_i = f_v f_w/(1 + w q_d r_s)$<br>$\beta_i$: stress factor of interception loss;<br>f_w: wet fraction of canopy; w: wind speed; q_d: surface drag coefficient; r_s: architectural resistance; ρ: air density; q_{sat}: saturated air humidity; q_a: air humidity; r_a: aerodynamic resistance.
Surface Externalisée	SURFEX	$E_i = \delta(q_{sat} - q_a)/r_a$<br>$\delta = \min(1, W_r/(0.125 f_v L))$<br>$\partial W_r/\partial t = f_v P - E_r - R_r$<br>$w_r$: water content of the interception reservoir; δ: percentage of liquid water on leaves; E_r: interception loss from the canopy reservoir; R_r: drainage rate from the canopy reservoir.

Supplementary Table 2. List of the 76 FLUXNET sites used in the analysis. Reported are coordinates (latitude and longitude), plant functional type (PFT, defined by the International Geosphere Biosphere Programme class), maximum leaf area index (LAI_{max}), Infra-red gas analyzers (IRGAs, 1: Closed path; 0: Open path), tower height (Tower_h) and canopy height (Canopy_h). NA, not available.

Code	Latitude	Longitude	PFT	LAI_{max} (unit: m^2 m^{-2})	IRGAs	Tower_h (unit: m)	Canopy_h (unit: m)
AU-Ade	-13.0769	131.1178	WSA	2.04	0	21	12.5
AU-DaS	-14.1593	131.3881	SAV	1.8768	0	21	16.4
AU-Dry	-15.2588	132.3706	SAV	1.66	0	15	12.3
AU-Gin	-31.3764	115.7138	WSA	1.4076	0	15	6.8
AU-How	-12.4943	131.1523	WSA	2.4	0	23	16
AU-RDF	-14.5636	132.4776	WSA	NA	0	21	16
AU-Rob	-17.1175	145.6301	EBF	NA	0	40	28
AU-Stp	-17.1507	133.3502	GRA	NA	0	5	1
AU-Tum	-35.6566	148.1517	EBF	NA	0	70	40.41
AU-Wac	-37.4259	145.1878	EBF	5.01	0	95	80
AU-Wom	-37.4222	144.0944	EBF	6.4124	0	30	22
BE-Bra	51.30761	4.51984	MF	2.1	1	41	21.22
BE-Lon	50.5516	4.7461	CRO	NA	1	3.21	0.48
BE-Vie	50.3051	5.9981	MF	5.1	1	44.7	33.57
BR-Sa3	-3.018	-54.9714	EBF	5.21	1	64	27.5
CA-Gro	48.2167	-82.1556	MF	4.3	0	43.3	31
CA-Obs	53.98717	-105.11779	ENF	3.41	1	25	11
CA-Qfo	49.6925	-74.3421	ENF	3.7	1	24	13.8
CA-SF1	54.485	-105.8176	ENF	3.4	0	12.1	6
CH-Dav	46.8153	9.8559	ENF	5.5	1	35	25
CN-Cng	44.5934	123.5092	GRA	NA	0	2	0.60
CN-Du2	42.0467	116.2836	GRA	NA	0	4	0.43
CN-Du3	42.0551	116.2809	GRA	NA	0	2	0.25

CN-HaM	37.37	101.18	GRA	2.8	0	2.2	0.3
DE-Geb	51.1001	10.9143	CRO	NA	1	3.61	0.33
DE-Kli	50.8931	13.5224	CRO	NA	1	3.5	0.46
DE-Lkb	49.0996	13.3047	ENF	NA	0	9	2
DE-Obe	50.7867	13.7213	ENF	NA	1	30	19.64
DE-RuS	50.86591	6.44714	CRO	NA	0	2.46	0.29
DE-Seh	50.8706	6.4497	CRO	NA	0	1.89	0.43
DE-Tha	50.9624	13.5652	ENF	7.6	1	42	28.21
DK-Fou	56.4842	9.58722	CRO	NA	0	2.62	0.94
ES-Amo	36.83361	-2.25232	OSH	NA	0	3.05	0.42
ES-LJu	36.92659	-2.75212	OSH	NA	0	2.5	0.5
FI-Hyy	61.8474	24.2948	ENF	6.7	1	32.8	14.98
FI-Let	60.64183	23.95952	ENF	NA	1	25.7	19.03
FR-Gri	48.8442	1.9519	CRO	NA	0	3.41	0.59
FR-LBr	44.7171	-0.7693	ENF	3.2	1	37	20.2
GH-Ank	5.26854	-2.69421	EBF	NA	1	50	27
IT-SR2	43.73202	10.29091	ENF	NA	1	24.3	19
IT-SRo	43.7279	10.2844	ENF	NA	1	22.1	18.7
MY-PSO	2.973	102.3062	EBF	6.52	0	54	35
NL-Loo	52.1666	5.7436	ENF	2.1	0	26	16.99
RU-Cok	70.82914	147.49428	OSH	1.5	0	4.7	0.5
RU-Fyo	56.4615	32.9221	ENF	3	1	29	25.41
US-AR1	36.4267	-99.42	GRA	NA	0	2.84	0.5
US-ARb	35.5497	-98.0402	GRA	NA	0	3.6	0.1
US-ARc	35.54649	-98.04	GRA	NA	0	4.15	0.4
US-ARM	36.6058	-97.4888	CRO	NA	0	3.8	0.19
US-Blo	38.8953	-120.6328	ENF	4.63	1	10.9	2.59
US-CRT	41.628495	-83.347086	CRO	NA	0	2	0.42
US-GBT	41.36579	-106.2397	ENF	3	0	27.15	11.55

US-GLE	41.3665	-106.2399	ENF	4.5	0	22.65	9.725
US-Lin	36.3566	-119.8423	CRO	NA	1	9.18	3.7
US-LWW	34.9604	-97.9789	GRA	NA	0	3	0.4
US-Me1	44.5794	-121.5	ENF	NA	0	10	7.2
US-Me2	44.4523	-121.5574	ENF	3.58	0	33	15.66
US-Me4	44.4992	-121.6224	ENF	2	0	47	16.5
US-Me5	44.43719	-121.56676	ENF	1.1	0	12	4.3
US-Me6	44.3233	-121.6078	ENF	1.8	0	12	5.855
US-MMS	39.3232	-86.4131	DBF	4.62	1	46	26
US-Ne1	41.1651	-96.4766	CRO	NA	1	4.54	1.59
US-Ne2	41.1649	-96.4701	CRO	NA	1	4.51	1.13
US-Ne3	41.1797	-96.4397	CRO	NA	1	4.48	0.97
US-NR1	40.0329	-105.5464	ENF	3.65	1	21.5	13.43
US-Prr	65.12367	-147.48756	ENF	0.73	1	11	2.53
US-Syv	46.242	-89.3477	MF	7.53	1	36	27.5
US-Ton	38.4316	-120.966	WSA	3.28	0	23.5	9.9
US-Tw2	38.1047	-121.6433	CRO	NA	0	5.15	1.81
US-Tw3	38.1159	-121.6467	CRO	NA	0	2.8	0.28
US-Twt	38.10872	-121.6531	CRO	NA	0	3.18	0.58
US-WCr	45.8059	-90.0799	DBF	5.36	1	29.6	24.3
US-Whs	31.7438	-110.0522	OSH	0.57	0	4.8	1
US-Wi1	46.73047	-91.23294	DBF	NA	0	9	6
US-Wi3	46.63472	-91.098667	DBF	3.9	0	26	20.5
US-Wkg	31.7365	-109.9419	GRA	0.9	0	4.8	0.5

Supplementary Figures

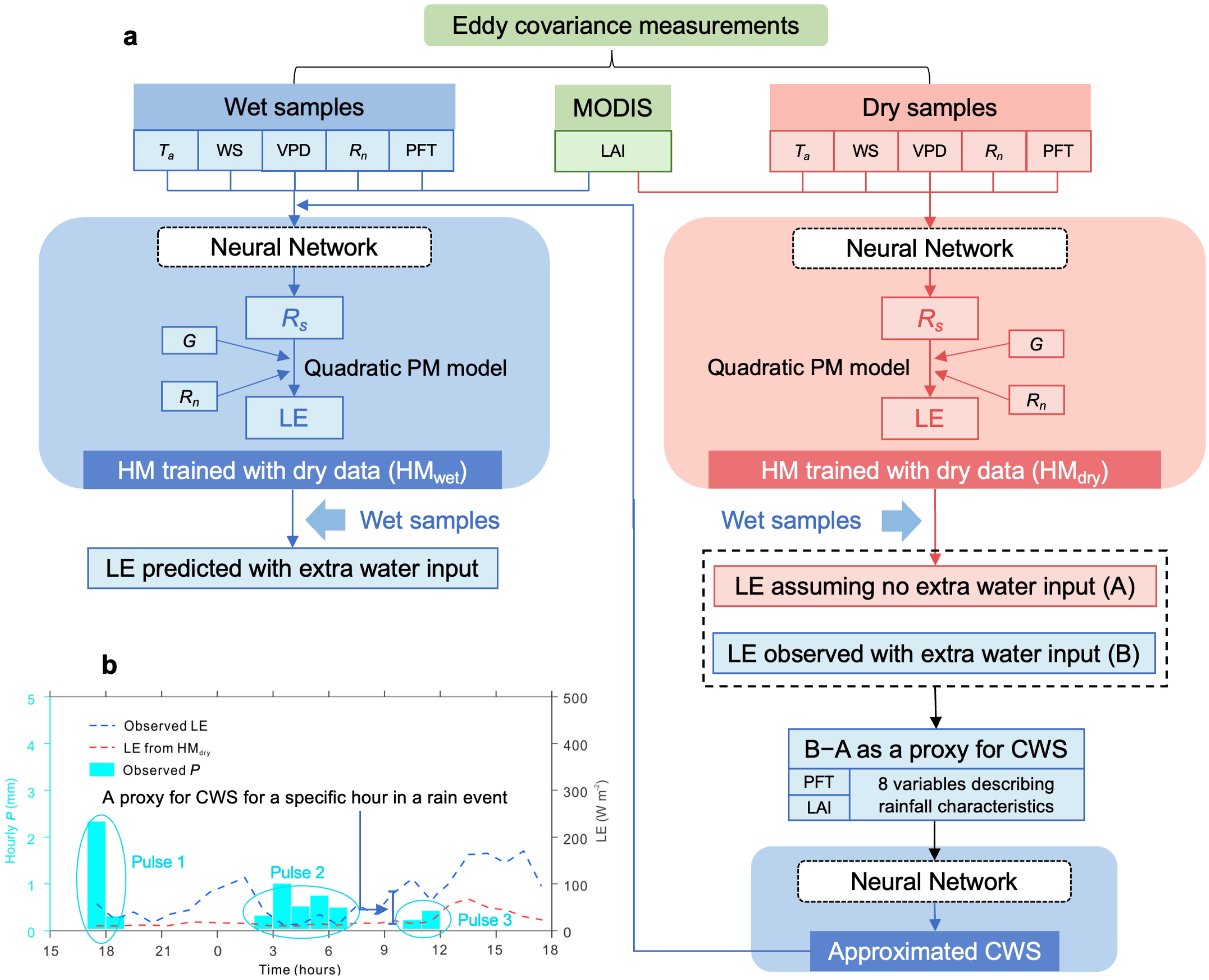

Supplementary Figure 1. The schematic of isolating rainfall interception loss (E_i) from tower-observed latent heat flux (LE). a, Major procedures of E_i prediction using two hybrid models, as described in "Hybrid model architecture, training and prediction" (Methods). **b**, An example for the characterization of canopy water storage (CWS) during a rain event. Cyan bars show hourly rainfall, which can be separated into individual rainfall pulses by the no-rain intervals. Blue and red curves show the LE observed at flux sites and estimated by hybrid models trained with wet samples (HM_{wet}) and dry samples (HM_{dry}), respectively. For any specific hour, the difference between the two curves is used as a measure of CWS, which is related to characteristics of the entire event and also of the latest pulse. P precipitation, R_s surface resistance, LAI leaf area index, R_n net solar radiation, VPD vapour pressure deficit, PFT plant functional types, G ground heat flux.

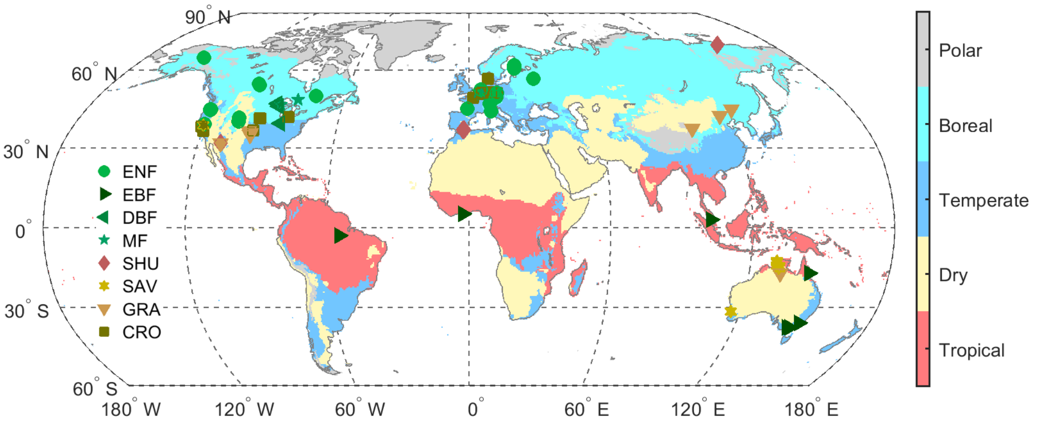

Supplementary Figure 2. The spatial distribution of the 76 FLUXNET sites used for analysis. Symbols indicate the eight plant functional types (defined by the International Geosphere Biosphere Programme class) of sites. The background map shows the five major global eco-climatic zones (tropical, dry, temperate, boreal and polar) according to the Köppen–Geiger climate classification. ENF evergreen needleleaf forest, EBF evergreen broadleaf forest, DBF deciduous broadleaf forest, MF mixed forest, SAV savanna, SHU shrubland, GRA grassland, CRO cropland.

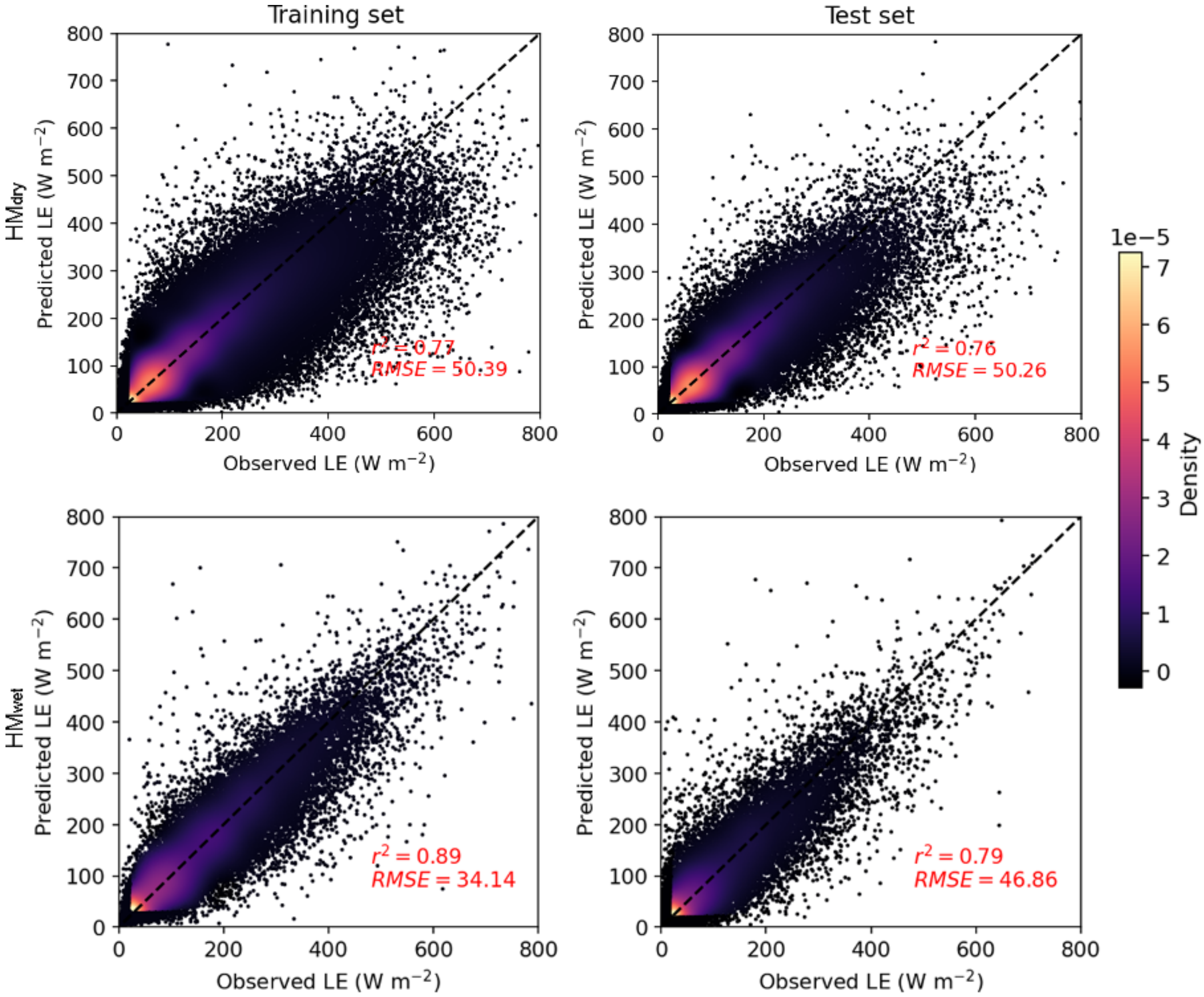

Supplementary Figure 3. Evaluation of model performance. Scatterplots of hourly latent heat flux (LE) predicted by hybrid models (y axis) against that observed at the flux sites (x axis). This comparison is conducted for HM_{wet} (**a, c**) trained with wet samples, and HM_{wet} (**b, d**) trained with dry samples, respectively. For both models, 80% of site hour samples are used for training, and the rest for test. The black dash lines show the identity line. RMSE root mean square error.

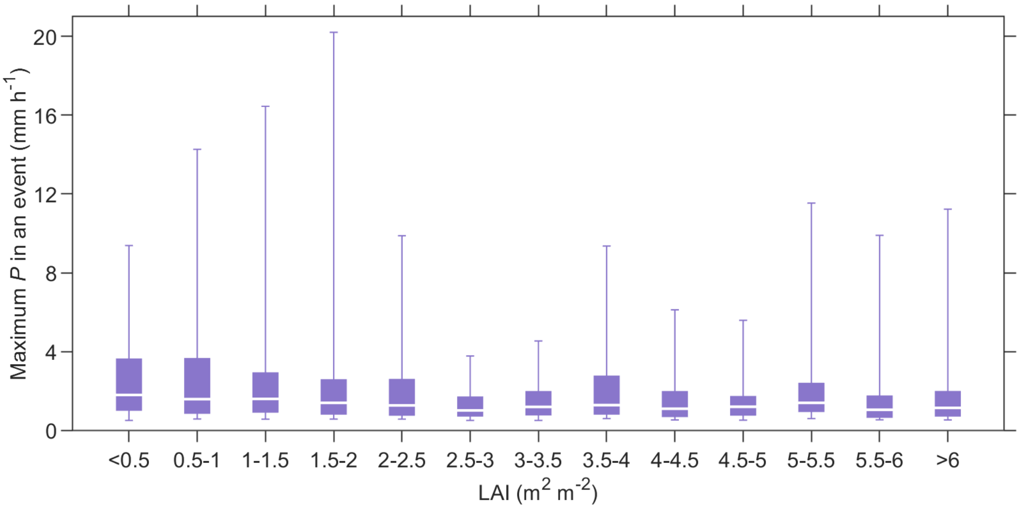

Supplementary Figure 4. Precipitation (*P*) variability for different levels of leaf area index (LAI). Boxplot shows the maximum *P* rate for all available rain events, across the gradient of LAI binned into 0.5 m^2 m^{-2} intervals. Each box plot shows the median (white line), interquartile range (purple shading), and the 5th and 95th percentiles (whiskers).

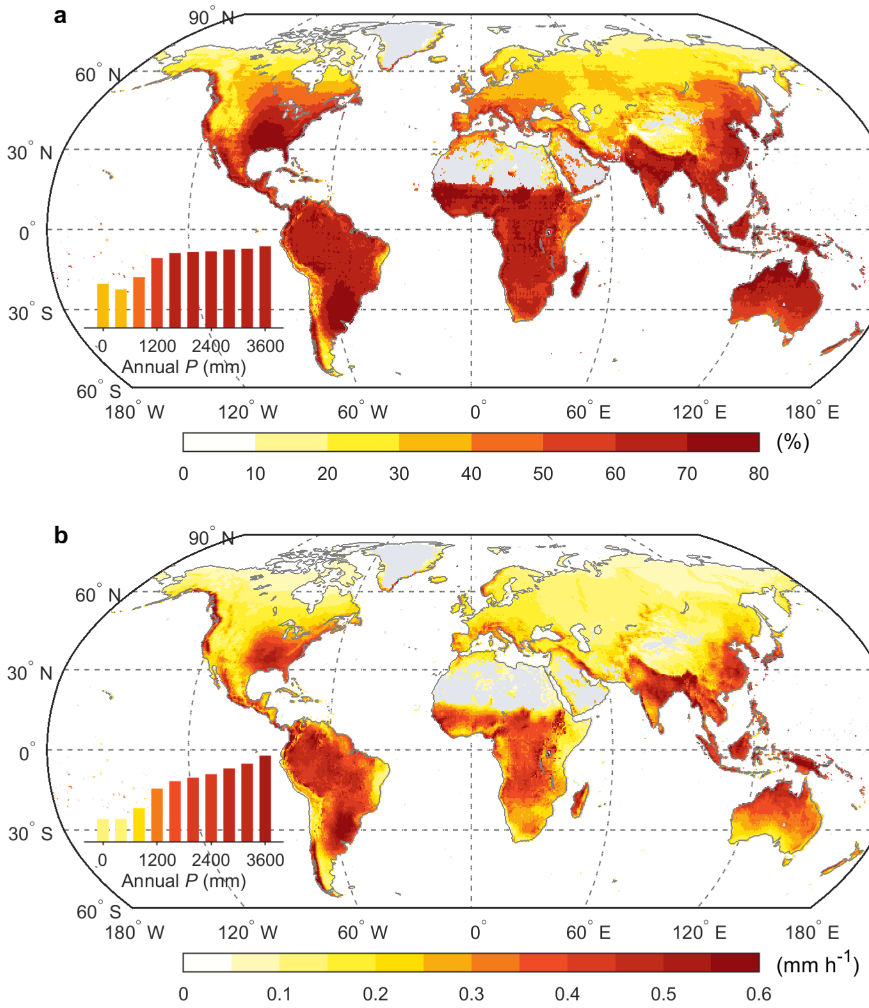

Supplementary Figure 5. Spatial variations of rainfall (*P*) intensity. a, the fraction of annual accumulated *P* from intensely raining hours ($P > 1$ mm h^{-1}) during 2000–2020. **b**, the average rainfall rates of all raining hours ($P > 0$ mm h^{-1}) during 2000–2020. The inset histogram shows the mean value across the average precipitation gradients.

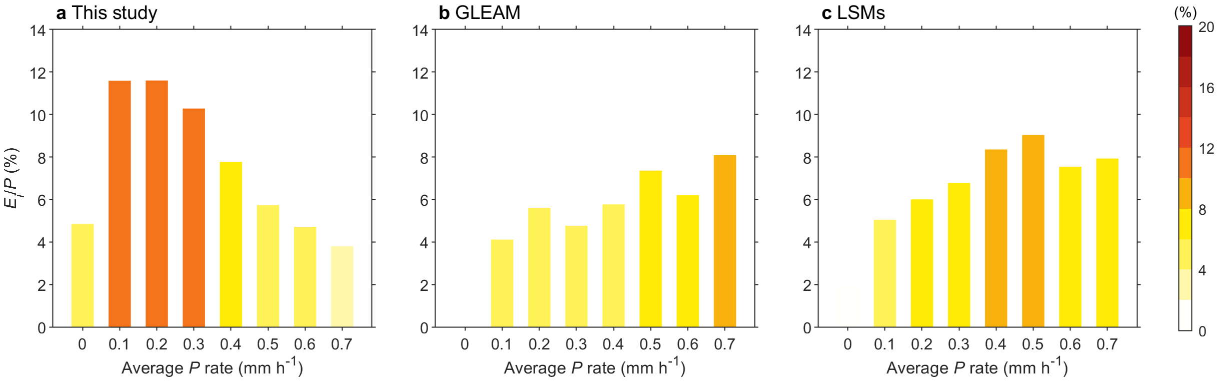

Supplementary Figure 6. Variations in the fraction of rainfall interception loss (E_i/P) across rainfall (P) intensity. The histograms show the destitution of E_i/P ratio from our data-driven result (**a**), GLEAM (**b**) and LSMs (**c**), across the spatial gradient of average rainfall rate (mm h^{-1}, shown in Fig. S8b).

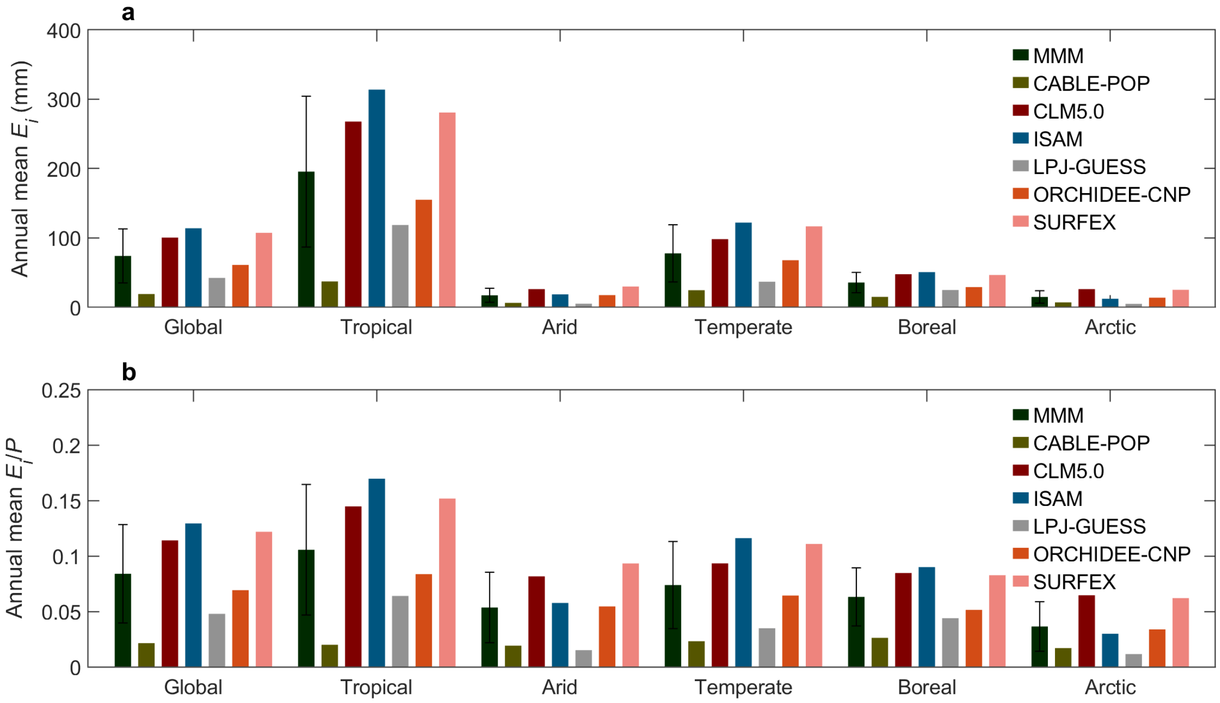

Supplementary Figure 7. Estimates of annual mean interception evaporation (E_i) and its fraction of rainfall (E_i/P) from land surface models (LSMs) grouped by climate zones. Black bars represent the multi-model mean value, and error bars represent the 1-SD among models. Estimates by each single LSM are in different colors.

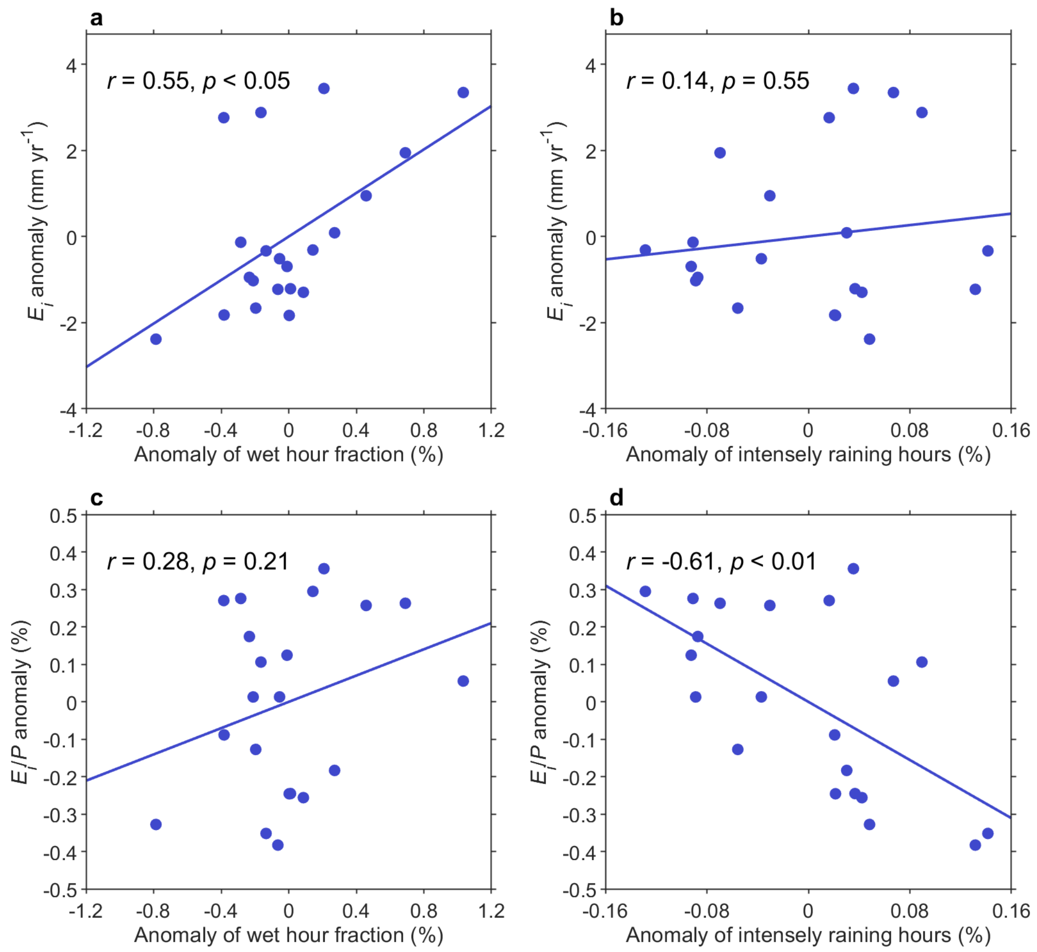

Supplementary Figure 8. Relationship between interception evaporation, E_i (or its fraction to total rainfall, E_i/P) and rainfall (P) characteristics. a, b, Scatterplots of annual mean E_i against the fraction of wet hours (**a**) and intensely raining hours (**b**), respectively. **c, d**, Scatterplots of annual E_i/P against the fraction of wet hours (**c**) and intensely raining hours (**d**), respectively. In **a**, the relationship is assessed both for values averaged globally, or for dry and wet regions separately (based on definitions using aridity index). All variables use inter-annual anomalies by subtracting the corresponding average during 2000−2020. Black lines show the best-fit regression. Labels show the Pearson correlation and its significance.

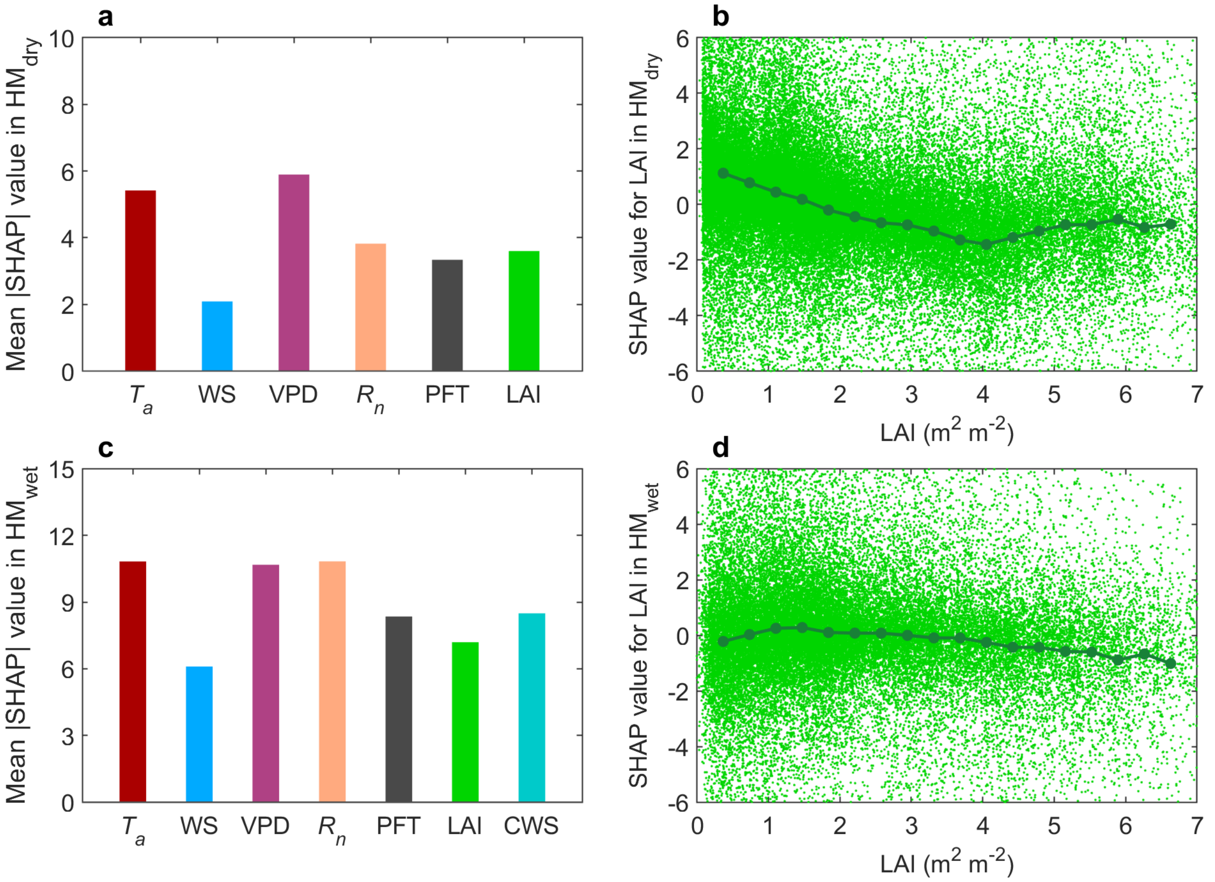

Supplementary Figure 9. Dependence of surface resistance (R_s) on predictors in hybrid models. a, **c**, the relative importance of predictors for variations of R_s in hybrid models trained with wet samples (HM_{wet}) and dry samples (HM_{dry}), respectively. The variable importance is assessed with the mean |SHAP| (SHapley Additive exPlanations) value. **b**, **d**, partial dependence plots of the SHAP value against LAI from HM_{dry} and HM_{wet}, respectively, where the dark green curve represents the median value along gradients of leaf area index (LAI). Note that R_s, instead of latent heat (LE), is presented here because R_s is the target directly simulated by the built-in NN of the hybrid model. The decreasing R_s along LAI in **b** and **d** indicate increasing LE along LAI gradients since R_s and LE are inversely related.

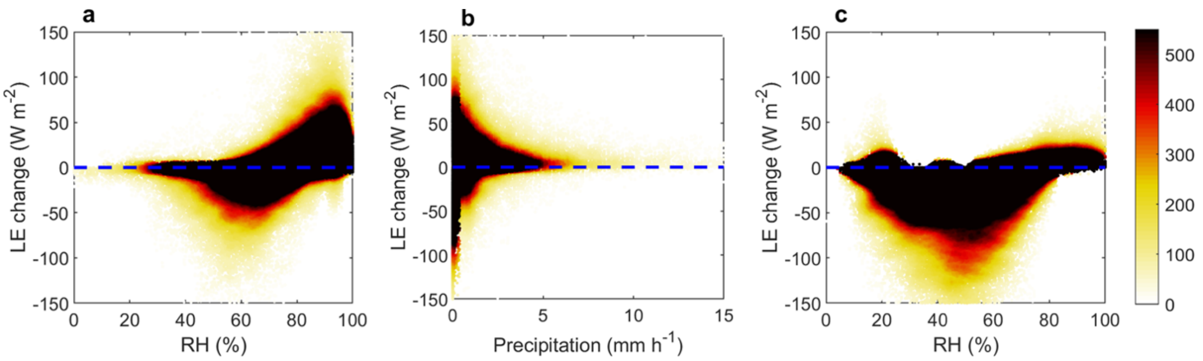

Supplementary Figure 10. Changes of hourly latent heat flux (LE) by correcting for an artificial dependence on relative humidity (RH) and rainfall. Heatmaps show the LE changes along the gradients of RH and precipitation amount. The shading shows the density of site-hour samples. The LE changes indicate the difference between the hypothesized LE under conditions of moderate RH (50%) and no rain (predicted via a neural network; Methods) and the original LE. **a** and **b** show the results for wet-hour LE, and **c** shows that for dry-hour LE.

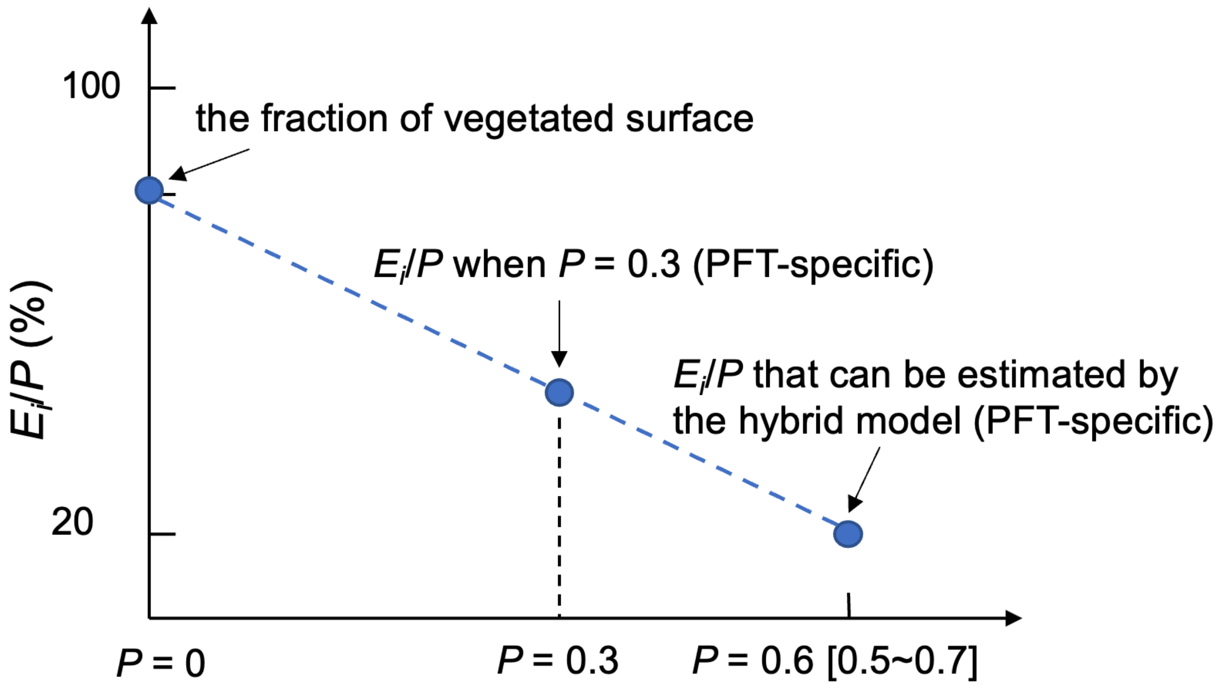

Supplementary Figure 11. Schematic of estimating the fraction of rainfall interception loss (E_i/P) in light rain (with hourly rainfall P < 0.5mm h^{-1}) conditions. In brief, for a specific rain rate within 0 and 0.5 mm h^{-1}, we linearly interpolated the E_i/P ratio based on the information known for 0 and 0.6 mm h^{-1} (Details in "Global E_i mapping driven by satellite and reanalysis data").
